# Validation of a tool to assess knowledge about immunization among people living with HIV/AIDS

**DOI:** 10.1590/0034-7167-2025-0199

**Published:** 2026-05-11

**Authors:** Larissa Gerin, Marcela Antonini, Elucir Gir, Renata Karina Reis

**Affiliations:** I Secretaria Municipal da Saúde, Divisão de Vigilância Epidemiológica. Ribeirão Preto, São Paulo, Brazil; II Universidade de São Paulo. Ribeirão Preto, São Paulo, Brazil

**Keywords:** HIV, Immunization, Education, Continuing, Knowledge, Vaccination Coverage., VIH, Inmunización, Educación Continua, Conocimiento, Cobertura de Vacunación.

## Abstract

**Objectives:**

to validate a tool that assesses healthcare professionals’ knowledge regarding immunization of people living with HIV/AIDS.

**Methods:**

a methodological validity study by experts in the field.

**Results:**

the tool, titled “*Vacina+HIV*”, consists of 35 questions about immunization of people living with HIV/AIDS. The tool was validated by 11 experts. Regarding the Confidence Validity Interval, the values achieved were 0.99 for content, 0.96 for objective, 1.00 for written language, and 0.98 for relevance, reaching an overall Confidence Validity Interval of 0.98, and being considered validated.

**Conclusions:**

this is believed to be the first validated tool on this topic for healthcare professionals in Brazil. The results demonstrate the validity of a tool that can help identify knowledge gaps that can be better addressed through different continuing education strategies.

## RESUMEN

**Objetivos:**

validar una herramienta que evalúa los conocimientos de los profesionales sanitarios sobre la inmunización de las personas con VIH/SIDA.

**Métodos:**

estudio de validación metodológica realizado por expertos en la materia.

**Resultados:**

la herramienta, denominada “Vacina+VIH”, consta de 35 preguntas sobre la inmunización de las personas con VIH/SIDA. La herramienta fue validada por 11 expertos. En cuanto al Intervalo de Validez de Confianza, los valores obtenidos fueron 0,99 para el contenido, 0,96 para el objetivo, 1,00 para el lenguaje escrito y 0,98 para la relevancia, alcanzando un Intervalo de Validez de Confianza general de 0,98 y considerándose validada.

**Conclusiones:**

se considera que esta es la primera herramienta validada sobre este tema para profesionales de la salud en Brasil. Los resultados demuestran la validez de una herramienta que puede ayudar a identificar brechas de conocimiento que pueden abordarse mejor mediante diferentes estrategias de educación continua.

## INTRODUCTION

With the advent and modernization of antiretroviral therapy (ART), the life expectancy of those living with the human immunodeficiency virus (HIV) has increasingly matched that of those living with any other chronic disease. In a reality where people living with HIV/AIDS (PLWHA) have seen their quality of life and life expectancy increase, it is crucial that disease prevention efforts be strengthened for this population^(1,2)^.

In this new scenario, with the aging of PLWHA, the prevention of diseases that can be avoided or have their burden reduced through vaccination becomes a high-impact public health action, since these individuals can develop more serious conditions after exposure to agents that cause various infections commonly acquired by these individuals^(2)^.

For these reasons, immunization guidelines around the world recommend a different vaccination schedule for PLWHA compared to the general population^(1,3)^.

In Brazil, the Ministry of Health offers, through the Brazilian National Immunization Program (In Portuguese, *Programa Nacional de Imunizações* - PNI), a vaccination schedule with 13 immunizers, available free of charge, for different vaccine-preventable infections, which are administered in the immunization rooms of Primary Health Care units and Reference Centers for Special Immunobiological agents (In Portuguese, *Centros de Referência para Imunobiológicos Especiais* - CRIE) of the Brazilian Health System (In Portuguese, *Sistema Único de Saúde* - SUS)^(4)^.

With the advancement of science in immunization, with the discovery of new vaccines and, in addition, the incorporation of an increasing number of vaccines into vaccination schedules, whether in the public or private sector, being a vaccinator requires constant updating, and has become an increasingly complex task for nursing professionals, in addition to being a challenge for the nurse supervising this service^(4,5)^.

The challenge is no different for medical professionals who monitor these individuals in referral services for clinical follow-up and should prescribe the indicated vaccines, but often do not do so because they are not sure about the vaccines and their updated schedules^(4,5)^.

A study conducted in Brazil that assessed healthcare professionals’ knowledge on this topic identified gaps in the knowledge of nursing professionals who worked in immunization rooms and in clinical monitoring services for PLWHA^(6)^.

The application of this knowledge assessment tool in the aforementioned study could show that professionals may have insufficient knowledge regarding the indicated vaccines and their schedules for PLWHA, and that assessment can help identify these gaps to propose specific intervention measures^(7)^. The study demonstrated that educational activities can effectively improve knowledge on this topic; however, there are gaps in literature regarding the existence of validated tools that assess knowledge on this specific topic^(6)^.

### Study relevance

This article is the product of a thesis^(8)^ deposited in the institutional repertoire (https://www.teses.usp.br/teses/disponiveis/22/22132/tde-16052023-083922/pt-br.php).

## OBJECTIVES

To validate a tool that assesses healthcare professionals’ knowledge regarding the immunization of PLWHA.

## METHODS

### Ethical aspects

This study was conducted in accordance with national and international ethics guidelines, and was approved by the Research Ethics Committee of the *Universidade de São Paulo, Escola de Enfermagem de Ribeirão Preto*. Informed Consent Form (ICF) was obtained from all individuals involved in the study through the Research Electronic Data Capture (REDCap) data collection platform.

### Study design, period and site

This is a methodological study, carried out in March 2025, which sought to validate a knowledge assessment tool in relation to content, objective, written language, and relevance by healthcare professionals who are experts in the subject online^(9)^.

The text of this manuscript was developed in accordance with STrengthening the Reporting of OBservational studies in Epidemiology recommendations.

### Population and sample, inclusion criteria

Validity of the knowledge assessment tool’s content was carried out by healthcare professionals considered experts, selected by convenience taking into account Fehring criteria^(10)^: experts in HIV, immunization or Primary Health Care.

Experts were identified through the *Lattes* Platform, websites of institutions related to the topics involved in the study, scientific events, and recommendations from previously identified professionals. Experts were selected for their recognized work in the research field or for their practical experience in the field, with a specialization, master’s degree, or doctoral degree in one of the topics.

There is no consensus in literature regarding the number of judges to invite, nor the number of judges to make up the final sample. In this study, an invitation was sent via email to 33 professionals who met the above criteria, of whom 11 participants were included, meeting the expected number for validity, which was between five and ten experts^(11)^.

Healthcare professionals received an invitation via an automated email sent by the REDCap platform. Experts received a link to the platform, which provided an explanatory text about the study objectives, risks, and benefits. After agreeing, participants who agreed to participate were directed via a specific link to the ICF and, later, to the online questionnaire to answer the validity questions.

### Study protocol

The “*Vacina+HIV - avaliação do conhecimento sobre imunização de pessoas que vivem com HIV/AIDS para profissionais de saúde*” (Vaccine+HIV - assessment of knowledge about immunization of people living with HIV/AIDS for healthcare professionals) tool was developed taking into account: the professional experience of the authors of this study; the epidemiological situation of the country at the time, which was experiencing a decline in vaccination coverage; the growth of anti-vaccine movements; and the technical content provided by the PNI^(4,12,13)^.

The aim was to begin with an assessment of the importance of vaccination as an effective public health measure for controlling diseases in the community, and then move on to the technical content of individual immunization itself, considering which aspects of the technical standard could generate doubts and confusion for the teams.

The “*Vacina+HIV - avaliação do conhecimento sobre imunização de pessoas que vivem com HIV/AIDS para profissionais de saúde*” tool is composed of:

1. Identification data - date of birth, sex, place of work, time spent in the workplace, time since graduation, education, graduate level, participation in previous theoretical training in immunization.
2. Items related to technical-scientific knowledge on immunization of PLWHA (n=35) classified into four knowledge categories (Chart 1), composed of affirmative sentences in which only one response option can be chosen: “agree”, “disagree” or “do not know”^(14,15)^.

**Chart 1:** Items of the “*Vacina+HIV*” tool according to the knowledge category, Ribeirão Preto, São Paulo, Brazil, 2025 (free translation)

Category 1: Importance of vaccination as a public health practice/significance of health promotion
1. The only healthcare professional responsible for prescribing vaccines to PLWHA is the infectious disease expert. 2. PLWHA have specific vaccination recommendations from the PNI. 3. Immunization room staff can administer to PLWHA only the vaccines prescribed by the infectious disease expert monitoring the patient. 4. Actively seeking out individuals with overdue vaccination schedules by immunization room staff and clinical follow-up services is an important step to ensure the completeness of the vaccination schedule. 5. Some diseases, such as measles, have already been controlled. Therefore, in the risk-benefit assessment, there is no need to administer the measles vaccine to PLWHA. 6. The PNI provides several vaccines through the SUS, and the country maintains good vaccination coverage in adults. 7. Many countries face problems with vaccine refusal, a complex phenomenon involving several factors. This phenomenon has been gaining ground in Brazil in recent years. 8. An individual who does not agree to complete the vaccination schedule but accepts some vaccines may be considered vaccine hesitant. 9. As more vaccines become available and widely used in vaccination programs, people become less concerned about the safety of vaccines and less likely to question the need for them. 10. Anti-vaccination movements began in the 19^th^ century with the use of the smallpox vaccine, the first vaccine developed, and have been gaining momentum in recent years due to their spread on social media. 11. A decline in vaccination coverage increases the incidence of preventable diseases and, consequently, the number of preventable deaths, which poses a risk to public health.
**Category 2: Basic concepts in immunization**
12. Combination vaccines are those in which an immunologically less potent product is added to another immunologically more potent product, thus achieving immunological potency characteristics that it previously lacked. 13. A conjugate vaccine is one composed of two or more antigens from different infectious agents in a single preparation. 14. In inactivated non-conjugated polysaccharide vaccines, immunity is short-lived (generally three to five years) because the immune response does not involve the stimulation of lymphocytes related to cellular immunity. 15. During pre-vaccination screening, possible contraindications should be investigated, such as the use of antibiotics, which is contraindicated with most vaccines. 16. It is recommended to postpone vaccination in cases of moderate or severe acute febrile illness until the condition improves. 17. The occurrence of adverse events must be reported, and any adverse event constitutes a contraindication for future doses.
**Category 3: Live attenuated and inactivated vaccines - indications for people living with HIV/AIDS**
18. The hepatitis A vaccine is made from an inactivated virus. 19. Live attenuated viral vaccines, prepared with live antigens, cannot be administered to PLWHA. 20. Inactivated vaccines are produced from inactivated whole microorganisms or microorganism particles, in addition to viral vector and messenger RNA, and are not capable of causing disease. 21. A 38-year-old healthcare user comes to receive the adult double viral and triple viral vaccines with a referral from the healthcare provider where he or she is being monitored. The healthcare provider in the immunization room verifies that the user received a dose of the meningococcal ACWY vaccine ten days ago and advises the patient to return in 20 days, as other vaccinations with an interval of less than 30 days are contraindicated. 22. Inactivated vaccines are generally not contraindicated for PLWHA. 23. A 30-day interval must be observed between inactivated vaccines when not administered on the same day. 24. Attenuated vaccines can only be administered to asymptomatic PLWHA with a LT-CD4+ count > 350 cells/mm^3^. 25. COVID-19 vaccines can be administered to PLWHA.
**Category 4: Vaccination schedule for HIV-infected adults**
26. The influenza vaccine is contraindicated for individuals with LT-CD4+ < 200 cells/mm^3^. 27. PLWHA are recommended to receive the 23-valent pneumococcal vaccine with a two-dose schedule spaced five years apart. 28. Administering the yellow fever vaccine to PLWHA is contraindicated in any situation. 29. The 23-valent pneumococcal vaccine should be administered to PLWHA starting eight weeks after receiving the pneumococcal 13 vaccine. 30. The monovalent varicella vaccine is indicated for all PLWHA. 31. The ACWY meningococcal vaccine vaccination schedule for PLWHA is two doses spaced 60 days apart. 32. After completing the hepatitis B vaccination schedule for PLWHA, serology testing is recommended to assess seroconversion 30 to 60 days after the last dose of the vaccine. If the serology results are negative, repeating the full schedule is recommended. 33. Vaccinating healthcare professionals and family members of PLWHA is a way to increase protection for this population. 34. The ideal time to start any vaccine for PLWHA is six months after starting ART. 35. The HPV vaccination schedule for PLWHA consists of three doses (0, 2, and 6 months) for those aged 9 to 45 years.

*PLWHA - person living with HIV/AIDS; SUS - Sistema Único de Saúde (Brazilian Health System); PNI - Programa Nacional de Imunizações (Brazilian National Immunization Program); RNA - ribonucleic acid; HIV - human immunodeficiency virus; ART - antiretroviral therapy.*

Experts answered questions regarding their identification, as well as questions regarding the tool’s content validity. The questions regarding the tool’s content validity (n=24) were divided into content (n=9), objective (n=5), written language (n=7), and relevance (n=3). For each item, a professional was asked to choose only one response on the Likert scale: “totally agree” (5), “agree” (4), “disagree” (3), “totally disagree” (2), or “do not know”^(16,17)^.

### Analysis of results and statistics

In the analysis to characterize experts, the absolute (n) and relative (%) frequencies of categorical variables and the mean and standard deviation of numerical variables were calculated using the Statistical Package for the Social Sciences version 29.

Based on professionals’ responses, the Content Validity Index (CVI) was calculated to validate the knowledge assessment tool. To do this, we considered the percentage of experts who agreed with each item assessed, using an agreement index of 80% or higher as the validity parameter. The CVI was calculated by adding the sum of the “totally agree” and “agree” responses from each expert on each item of the validity questionnaire, divided by the total number of responses. Items rated “disagree” and “totally disagree” were revised according to judges’ suggestions^(15,18)^.

## RESULTS

To validate the tool for assessing knowledge on immunization of PLWHA for healthcare professionals, invitations were sent by email to 33 experts, with 11 experts responding within the stipulated period of 15 days (33.3% adherence).

In general, experts were predominantly female (81.8%; n=9). The mean age among experts who completed the validity stage (n=11) was 53.2 years (minimum: 38.6; maximum: 68.9; SD +/- 10.1), ranging from 30 to 69 years, with the majority in the age groups between 40 and 49 years (36.4%; n=4), and between 60 and 69 years (36.4%; n=4).

Concerning evaluators’ training, 54.5% (n=6) were nurses, and 45.5% (n=5) were physicians. Regarding the maximum graduate level, 9.0% (n=1) had a specialization; 45.5% (n=5) had a master’s degree; and 45.5% (n=5) had a doctoral degree. The mean time since training was 29.9 years (minimum: 15; maximum: 44; SD +/- 10.1).

As for the CVI, the values achieved were 0.99 for content, 0.96 for objective, 1.00 for written language and 0.98 for relevance. Thus, the knowledge assessment tool was considered validated regarding its content, achieving an overall CVI of 0.98 (Table 1).

**Table 1:** Content Validity Index of the “Vacina+HIV - avaliação do conhecimento sobre imunização de pessoas que vivem com HIV/AIDS para profissionais de saúde” (Vaccine+HIV - assessment of knowledge about immunization of people living with HIV/AIDS for healthcare professionals) tool, Ribeirão Preto, São Paulo, Brazil, 2025 (N=11)

	Totally agree	Agree	Disagree	Totally disagree	Do not know	CVI
**Content**						0,99
The content presented in the knowledge assessment tool corresponds to the proposed objectives.	7	4	0	0	0	1,00
The content assessed follows a logical sequence.	8	3	0	0	0	1,00
The content covered is in line with current Ministry of Health recommendations.	9	2	0	0	0	1,00
The questions presented are appropriate for the target audience.	6	5	0	0	0	1,00
The topic of the knowledge assessment tool is current.	10	1	0	0	0	1,00
The question wording is clear.	8	2	0	1	0	0,91
The questions are unambiguous.	8	3	0	0	0	1,00
The number of questions in the knowledge assessment tool is adequate.	7	4	0	0	0	1,00
The questions focus on relevant topics.	10	1	0	0	0	1,00
**Objective**						0,96
The knowledge assessment tool is suitable for assessing healthcare professionals’ knowledge regarding immunization of people living with HIV/AIDS.	4	6	1	0	0	0,91
The objectives are appropriate for implementation.	6	5	0	0	0	1,00
The knowledge assessment tool addresses the proposed topic.	8	3	0	0	0	1,00
The knowledge assessment tool is suitable for the teaching-learning process.	4	7	0	0	0	1,00
The knowledge assessment tool encourages reflection regarding vaccination of people living with HIV/AIDS.	8	2	1	0	0	0,91
**Language**						1,00
The language used is accessible to the target audience.	8	3	0	0	0	1,00
The language is appropriate for the knowledge assessment tool.	10	1	0	0	0	1,00
The verbal language is easy to understand.	6	5	0	0	0	1,00
The statements are presented clearly and objectively.	8	3	0	0	0	1,00
The knowledge assessment tool does not contain any errors or misleading information.	8	3	0	0	0	1,00
The question statements are objective.	8	3	0	0	0	1,00
The statements do not use absolute terms (always/never) or vague terms (rarely/usually).	9	2	0	0	0	1,00
**Relevance**						0,98
The knowledge assessment tool addresses important aspects of understanding the aspects involved in PLWHA vaccination.	9	2	0	0	0	1,00
The knowledge assessment tool encourages learning.	7	3	1	0	0	0,91
The knowledge assessment tool helps assess knowledge regarding PLWHA vaccination.	8	2	1	0	0	0,94
**Overall CVI**						0,98

*CVI - Content Validity Index; PLWHA - person living with HIV/AIDS.*

In relation to the content, experts suggested including more questions discussing practical cases, such as question 21. We chose not to include more practical cases because we understand that behaviors may differ depending on the location and that practical cases could be developed by the teams during training activities, taking these differences into account.

A change was recommended to the text of questions 9, 10, 20, 21, 32, and 35. To make the statement more complete and clearer, most suggestions were accepted, and statements were rewritten in accordance with the suggestions received.

It was pointed out that some questions, such as questions 8, 12, 13, and 14, were too difficult for mid-level professionals or those who did not work directly with the topic. Since the tool is intended for use only by professionals working with the topic, and the CVI for content-related questions was almost entirely 1.00, we chose to keep the questions as presented to experts.

When assessing the tool’s objective and relevance, experts considered it important and useful, but emphasized that it should be used as part of an educational activity, being one of the technologies used in association with others that encourage the discussion of cases and doubts.

Experts highlighted the importance of encouraging professionals to seek additional knowledge from other sources of information, in addition to using other strategies in continuing education activities, such as case studies, practical discussions, skills observation, or formative assessments, which would allow for a more comprehensive analysis of the knowledge and skills of the professionals involved.

The importance of discussing with professionals the organization of CRIE in their locality was also emphasized so that they understand where and how the immunobiological agents offered in a special way can be accessed.

The suggestions presented by experts helped ensure that the assessment tool “*Vacina+HIV - avaliação do conhecimento sobre imunização de pessoas que vivem com HIV/AIDS para profissionais de saúde*” (Vaccine+HIV - assessment of knowledge about immunization of people living with HIV/AIDS for healthcare professionals), based on the modifications made, was made available in a clearer format for use by teams without errors in the assessment of technical-scientific knowledge.

## DISCUSSION

PLWHA are at higher risk of complications and death from vaccine-preventable diseases. Therefore, there are special vaccination recommendations^(2)^.

Given the complexity of the vaccination schedule recommended for this population, healthcare professionals’ conduct, who play an essential role in prescribing vaccines and assessing the vaccination schedule completeness, may contribute to not achieving the desired vaccination rates.

Studies indicate that vaccination rates for PLWHA are below expectations for most vaccines recommended for this population. The reasons for not being vaccinated are multifactorial, ranging from issues related to individuals being vaccinated, healthcare services, and staff, to vaccine availability^(7,16,19,20)^.

Healthcare professionals play a central role in assessing individuals’ vaccination status and prescribing vaccines according to established protocols. Since these protocols are complex and undergo successive modifications, professionals must receive continuing training^(5)^.

The recommendation of vaccination by healthcare professionals has a positive association with vaccination rates, as it increases awareness of the importance of prescribing immunizations, especially for higher-risk groups. However, this is a topic little addressed in physician and nursing staff training^(21)^.

The first category of the knowledge assessment tool assesses how professionals understand vaccination as an important public health practice for disease control.

In recent years, the success of immunization programs, through the reduction and even eradication of diseases, has contributed to a decrease in the perception of risk and an increase in vaccine insecurity, in a scenario where fear of adverse events outweighs fear of the disease and its potential consequences. Vaccine hesitancy, despite being a recently coined term, has been growing worldwide, including among healthcare professionals, and should be included in immunization training content to encourage teams to combat it^(13,22)^.

Basic immunization concepts are assessed in the second category of the knowledge assessment tool, which covers complex terms such as the definition of combined, conjugated, and polysaccharide vaccines. Topics such as these may be unfamiliar or cause confusion among immunization professionals, which can lead to false contraindications and missed vaccination opportunities.

These terms need to be constantly reinforced in continuing education activities so that they are definitively absorbed among professionals, with greater reinforcement, especially among mid-level professionals, who may have a greater degree of difficulty understanding these terms and their application in daily work practice during screening in an immunization room^(5,19,20)^.

The third category of the tool addresses attenuated and inactivated vaccines and their indications for PLWHA. Administration of attenuated vaccines is contraindicated in the presence of severe immunosuppression, and this may lead to a false contraindication of immunizations for PLWHA^(5)^.

Virtually all PLWHA on ART with good adherence achieve viral suppression and are not immunosuppressed. Therefore, they have no contraindications to receiving vaccines containing live agents. Therefore, teams need to be confident about administering these vaccines, and a physician’s prescription helps significantly ensure this safety^(5,20)^.

Working with the topic of immunization of PLWHA among healthcare professionals requires that they have well-established in their knowledge framework, in addition to concepts related to immunology, the difference between living with HIV and being immunosuppressed.

It is necessary to revisit the basic concepts of HIV, immunosuppression, and ART so that, later, they can continue comprehensive care, which includes the vaccination schedule.

The final category of the knowledge assessment tool addresses the vaccination schedule for HIV-infected adults, discussing schedules for some recommended vaccines and the best time to administer the vaccines, in addition to prevention through vaccination of household contacts.

These questions are not exhaustive. Continuing education activities should include comprehensive coverage of all vaccines and their schedules, contraindications, and the specificities of each location (where vaccines are offered and how they can be accessed).

When using the tool validated in this study, we suggest that knowledge on the topic be assessed by the percentage of correct answers per 20% interval (insufficient (0 -| 20); regular (20 -| 40); good (40 -| 60); great (60 -| 80); excellent (80 -| 100)), as proposed by other authors^(23)^.

### Study limitations

We understand that the knowledge assessment tool used in isolation, outside of educational activities, may not be effective in increasing professionals’ knowledge regarding immunization of PLWHA; therefore, we recommend that it be used as part of these activities, to assess professionals’ prior knowledge.

Furthermore, it can be used after a training activity to assess whether the objective was achieved.

Another limitation of this study is the relatively small sample of experts (n=11) and a convenience sample, which may bias the results. Despite this, we believe that the tool validated in this study remains a valuable tool that can assist teams in continuing education activities on this topic, identifying knowledge gaps that need to be addressed.

### Contributions to nursing, health or public policy

The results presented in this study contribute to providing teams with a tool that can be used in continuing health education activities, helping to identify knowledge gaps that can be better addressed through different training strategies. All items assessed were considered “totally adequate” or “adequate” by most experts, demonstrating that the objective of updating and validating a knowledge assessment tool to make it available for use in educational activities nationwide was achieved. The tool developed and validated in this study is one of the technologies that can be used during healthcare professional training on this topic, also contributing to improving vaccination rates among PLWHA and, consequently, improving these individuals’ quality of life and life expectancy.

This validated tool can be used by healthcare service teams with immunization rooms and in healthcare services that provide care to PLWHA to identify gaps in the knowledge of professionals in these services.

By identifying these gaps, continuing team education initiatives can be developed to improve professionals’ knowledge on this topic, which could directly help increase vaccination rates for PLWHA.

## CONCLUSIONS

The relevance of this study is highlighted, since no validated knowledge assessment tool on immunization of PLWHA was found in the literature. Therefore, this is believed to be the first validated knowledge assessment tool on this topic aimed at healthcare professionals in Brazil.

We conclude that the “*Vacina+HIV - avaliação do conhecimento sobre imunização de pessoas que vivem com HIV/AIDS para profissionais de saúde*” (Vaccine+HIV - assessment of knowledge about immunization of people living with HIV/AIDS for healthcare professionals) tool can assist teams involved in continuing education activities and that unaddressed content and issues related to practice in each location should be included in other educational strategies used.

## Data Availability

The research data are available within the article.
