# Supplementary material for: Validation of a tool to assess knowledge about immunization among people living with HIV/AIDS
Source: Rev Bras Enferm. 2026 May 11;79:e20250199. doi: 10.1590/0034-7167-2025-0199 (PMC13169633; doi:10.1590/0034-7167-2025-0199)
Supplement: Translation

# Translation: Validação de ferramenta para avaliação do conhecimento sobre imunização de pessoas vivendo com HIV/AIDS

**DOI:** 10.1590/0034-7167-2025-0199pt

**Authors:** Larissa Gerin, Marcela Antonini, Elucir Gir, Renata Karina Reis

**Affiliations:** I Secretaria Municipal da Saúde, Divisão de Vigilância Epidemiológica. Ribeirão Preto, São Paulo, Brasil; II Universidade de São Paulo. Ribeirão Preto, São Paulo, Brasil

**Keywords:** HIV, Imunização, Educação Permanente, Conhecimento, Cobertura Vacinal.

## Abstract

**Objetivos:**

validar uma ferramenta que avalia o conhecimento de profissionais de saúde a respeito da imunização de pessoas que vivem com HIV/AIDS.

**Métodos:**

estudo metodológico de validação por especialistas na temática.

**Resultados:**

a ferramenta foi intitulada “Vacina+HIV”, constituindo-se de 35 questões sobre imunização de pessoas que vivem com HIV/AIDS. A ferramenta foi validada por 11 especialistas. Em relação ao Intervalo de Validade de Confiança, os valores alcançados foram 0,99 para conteúdo, 0,96 para objetivo, 1,00 para linguagem escrita e 0,98 para relevância, atingindo um Intervalo de Validade de Confiança geral de 0,98, sendo considerada validada.

**Conclusões:**

acredita-se que esta seja a primeira ferramenta validada sobre o tema voltado para profissionais de saúde no Brasil. Os resultados mostram evidências de validade de uma ferramenta que pode contribuir para identificar lacunas no conhecimento que podem ser melhor abordadas em diferentes estratégias de educação permanente.

## INTRODUÇÃO

A partir do advento e modernização da terapia antirretroviral (TARV), a expectativa de vida de quem vive com o vírus da imunodeficiência humana (HIV) tem se equiparado cada vez mais à daqueles que vivem com qualquer outra doença crônica. Em uma realidade onde as pessoas que vivem com HIV/AIDS (PVHA) tiveram a sua qualidade e expectativa de vida aumentadas, é de suma importância que as ações de prevenção de doenças sejam fortalecidas para este público^(1,2)^.

Neste novo cenário, com o envelhecimento das PVHA, a prevenção de doenças que podem ser evitadas ou ter a sua carga diminuída por meio da vacinação se torna uma ação de saúde pública de alto impacto, uma vez que esses indivíduos podem evoluir de forma mais grave após a exposição aos agentes que causam diversas infecções adquiridas comumente por esses indivíduos^(2)^.

Por esses fatores, as diretrizes de imunização do mundo todo recomendam um esquema de vacinação diferenciado para as PVHA em comparação com a população geral^(1,3)^.

No Brasil, o Ministério da Saúde oferece, por meio do Programa Nacional de Imunizações (PNI), um calendário de vacinação com 13 imunizantes, disponibilizados de forma gratuita, para diferentes infecções imunopreveníveis, que são administradas nas salas de vacinas das unidades de Atenção Primária à Saúde e dos Centros de Referência para Imunobiológicos Especiais (CRIE) do Sistema Único de Saúde (SUS)^(4)^.

A partir do avanço da ciência na imunização, com a descoberta de novos imunizantes e, além disso, a incorporação de um número cada vez maior de vacinas nos calendários de vacinação, seja no setor público ou privado, ser um vacinador exige atualização constante, e tem se tornado uma ação cada vez mais complexa para os profissionais de enfermagem, além de ser um desafio ao enfermeiro supervisor desse serviço^(4,5)^.

O desafio não é diferente para os profissionais médicos que acompanham esses indivíduos nos serviços de referência para acompanhamento clínico e deveriam prescrever as vacinas indicadas, mas muitas vezes não o fazem por não estarem seguros quanto às vacinas e aos seus esquemas atualizados^(4,5)^.

Estudo realizado no Brasil que avaliou o conhecimento de profissionais de saúde a respeito desta temática, identificou lacunas no conhecimento de profissionais de enfermagem que atuavam em salas de vacinação e nos serviços de acompanhamento clínico de PVHA^(6)^.

A aplicação da ferramenta de avaliação do conhecimento no estudo acima mencionado pôde evidenciar que os profissionais podem ter um conhecimento insuficiente a respeito das vacinas indicadas e seus esquemas para PVHA, e que a avaliação pode auxiliar na identificação dessas lacunas para propor medidas interventivas específicas^(7)^. O estudo demonstrou que atividades educativas podem efetivamente melhorar o conhecimento nesta temática, porém há lacunas na literatura sobre a existência de ferramentas validadas que avaliem o conhecimento nessa temática específica^(6)^.

### Relevância do estudo

Este artigo é produto de tese^(8)^ depositada no repertório institucional (https://www.teses.usp.br/teses/disponiveis/22/22132/tde-16052023-083922/pt-br.php).

## OBJETIVOS

Validar uma ferramenta que avalia o conhecimento de profissionais de saúde a respeito da imunização de PVHA.

## MÉTODOS

### Aspectos éticos

O estudo foi conduzido de acordo com as diretrizes de ética nacionais e internacionais, e aprovado pelo Comitê de Ética em Pesquisa da Escola de Enfermagem de Ribeirão Preto da Universidade de São Paulo. O Termo de Consentimento Livre e Esclarecido (TCLE) foi obtido de todos os indivíduos envolvidos no estudo por meio da plataforma de coleta de dados *Research Electronic Data Capture* (REDCap).

### Desenho, período e local do estudo

Trata-se de estudo metodológico, realizado em março de 2025, que buscou validar uma ferramenta de avaliação do conhecimento em relação ao conteúdo, objetivo, linguagem escrita e relevância por profissionais de saúde *experts* na temática de forma *online* ^(9)^.

O texto deste manuscrito foi desenvolvido de acordo com as recomendações do *STrengthening the Reporting of OBservational studies in Epidemiology*.

### População e amostra, critérios de inclusão

A validação do conteúdo da ferramenta de avaliação do conhecimento foi realizada por profissionais de saúde considerados *experts*, selecionados por conveniência levando em consideração os critérios de Fehring^(10)^: especialistas na temática HIV, imunização ou Atenção Primária à Saúde.

Os *experts* foram identificados através da Plataforma *Lattes*, de sites de instituições relacionadas às temáticas envolvidas no estudo, em eventos científicos e por indicações de profissionais previamente identificados. Os *experts* foram selecionados por serem reconhecidos em sua atuação no campo da pesquisa ou por sua prática na área, com especialização, mestrado ou doutorado em uma das temáticas.

Não há consenso na literatura quanto ao número de juízes a serem convidados, nem do número de juízes a integrar a amostra final. Neste estudo, o convite foi encaminhado via e-mail para 33 profissionais que atendiam aos critérios acima, dos quais 11 participantes foram incluídos, o que atendeu ao número esperado para validação que era entre cinco e dez *experts* ^(11)^.

Os profissionais de saúde receberam um convite por meio de e-mail automático encaminhado pela plataforma REDCap. Os *experts* receberam um *link* que direcionava para a plataforma, que fornecia um texto explicativo sobre os objetivos, riscos e benefícios da pesquisa. Após concordância, os participantes que aceitaram participar do estudo foram direcionados por meio de *link* específico ao TCLE e, posteriormente, ao questionário *online* para responder às questões referentes à validação.

### Protocolo do estudo

A ferramenta “Vacina+HIV - avaliação do conhecimento sobre imunização de pessoas que vivem com HIV/AIDS para profissionais de saúde” foi elaborada levando em consideração: a experiência profissional dos autores deste estudo; a situação epidemiológica do país no momento, que vivenciava a queda nas coberturas vacinais; o crescimento dos movimentos antivacina; e o conteúdo técnico disponibilizado pelo PNI^(4,12,13)^.

Pretendeu-se partir de uma avaliação da importância do ato de vacinar quanto medida eficaz de saúde pública para controlar as doenças na comunidade, até chegar ao conteúdo técnico em si da imunização individual, pensando em quais conteúdos da norma técnica poderiam gerar dúvidas e confusões para as equipes.

A ferramenta “Vacina+HIV - avaliação do conhecimento sobre imunização de pessoas que vivem com HIV/Aids para profissionais de saúde” é composta por:

1. Dados de identificação - data de nascimento, sexo, local de trabalho, tempo de atuação no local de trabalho, tempo de formação, formação, nível de pós-graduação, participação em treinamento teórico em imunização anteriormente.
2. Itens referentes ao conhecimento técnico-científico em imunização de PVHA (n=35) classificados em quatro categorias de conhecimento (Quadro 1), compostos por frases afirmativas em que apenas uma opção de resposta pode ser escolhida: “concordo”, “discordo” ou “não sei”^(14,15)^.

**Quadro 1:** Itens da ferramenta “Vacina+HIV” de acordo com a categoria de conhecimento, Ribeirão Preto, São Paulo, Brasil, 2025

Categoria 1: Importância da vacinação como prática de saúde pública/significado da promoção da saúde
1. O único profissional de saúde responsável por indicar vacinas às PVHA é o infectologista. 2. As PVHA têm recomendações de vacinação específicas pelo PNI. 3. A equipe da sala de vacinação pode administrar na PVHA somente as vacinas que forem prescritas pelo médico infectologista que realiza o acompanhamento do paciente. 4. A busca ativa de indivíduos com esquema de vacinação em atraso pela equipe das salas de vacinação e dos serviços de acompanhamento clínico se constitui em uma importante ação para garantir a completude do esquema vacinal. 5. Algumas doenças já foram controladas, como o sarampo. Assim, na avaliação de risco e benefício, não há a necessidade de administrar a vacina contra sarampo em PVHA. 6. O PNI disponibiliza vários imunizantes através do SUS, e o país mantém boas coberturas vacinais em indivíduos adultos. 7. Muitos países enfrentam problemas com a recusa vacinal, um fenômeno complexo que envolve vários fatores. Este fenômeno vem se estabelecendo no Brasil nos últimos anos. 8. O indivíduo que não concorda em completar o esquema vacinal porém aceita receber algumas vacinas pode ser considerado um hesitante quanto à vacinação. 9. À medida que mais vacinas são disponibilizadas e amplamente utilizadas em programas de vacinação, as pessoas passam a se preocupar menos com a segurança dos imunizantes e a questionar menos a necessidade de sua aplicação. 10. Os movimentos antivacinais têm seu início no século XIX, a partir da utilização da vacina contra a varíola, a primeira vacina desenvolvida, e vem ganhando forças nos últimos anos por sua propagação nas mídias sociais. 11. A queda nas coberturas vacinais aumenta a incidência de doenças evitáveis e, consequentemente, aumenta o número de mortes evitáveis, o que se constitui um risco para a saúde pública.
**Categoria 2: Conceitos básicos em imunização**
12. Vacinas combinadas são aquelas em que um produto imunologicamente menos potente é acrescentado a outro produto imunologicamente mais potente, conseguindo-se, dessa maneira, que o primeiro produto adquira características de potência imunológica que antes não possuía. 13. Vacina conjugada é aquela composta por dois ou mais antígenos de agentes infecciosos diferentes em uma única preparação. 14. Nas vacinas inativadas polissacarídicas não conjugadas, a imunidade é de curta duração (três a cinco anos, em geral), pois a resposta imune não envolve a estimulação de linfócitos relacionados à imunidade celular. 15. Na triagem pré-vacinação, devem ser investigados possíveis contraindicações, como o uso de antibióticos, que contraindica a maioria das vacinas. 16. Recomenda-se adiar a vacinação em caso de doença febril aguda moderada ou grave até a melhora do quadro. 17. A ocorrência de eventos adversos deve ser notificada, e qualquer evento adverso se constitui em contraindicação para doses futuras.
**Categoria 3: Vacinas atenuadas e inativadas - indicações para pessoas vivendo com HIV/AIDS**
18. A vacina contra hepatite A é composta por vírus inativado. 19. As vacinas virais vivas atenuadas, preparadas com antígenos vivos, não podem ser administradas em PVHA. 20. As vacinas inativadas são produzidas a partir de microrganismos inteiros inativados ou partículas de microrganismos, além de vetor viral e RNA mensageiro, e não são capazes de produzir doenças. 21. Um usuário do serviço de saúde, com 38 anos de idade, comparece para receber as vacinas dupla adulto e tríplice viral com encaminhamento do profissional do serviço onde realiza o seu acompanhamento clínico. A profissional da sala de vacinas verifica que o usuário recebeu uma dose de vacina meningocócica ACWY há dez dias, e orienta o paciente a retornar em 20 dias, pois está contraindicado fazer outras vacinas com intervalo inferior a 30 dias. 22. As vacinas inativadas geralmente não são contraindicadas para PVHA. 23. É preciso respeitar 30 dias de intervalo entre as vacinas inativadas quando não aplicadas no mesmo dia. 24. As vacinas atenuadas somente podem ser administradas em PVHA assintomáticas com a contagem de LT-CD4+ > 350 céls/mm^3^. 25. As vacinas contra Covid-19 podem ser administradas em PVHA.
**Categoria 4: Esquema vacinal para adultos infectados pelo HIV**
26. A vacina contra *influenza* é contraindicada para pessoas com LT-CD4+ < 200 céls/mm^3^. 27. PVHA têm a recomendação de receber a vacina pneumocócica 23-valente com esquema de duas doses com intervalo de cinco anos entre elas. 28. É contraindicado administrar a vacina contra febre amarela em PVHA em qualquer situação. 29. A vacina pneumocócica 23-valente deve ser administrada nas PVHA a partir de oito semanas da data de recebimento da vacina pneumocócica 13. 30. A vacina monovalente contra varicela é indicada para todas as PVHA. 31. O esquema de vacinação com a vacina meningocócica ACWY para PVHA é de duas doses com intervalo de 60 dias. 32. Após a conclusão do esquema de vacinação contra hepatite B em PVHA, é recomendada a realização de sorologia para avaliar a soroconversão de 30 a 60 dias após a última dose da vacina. Caso a sorologia tenha resultado negativo, recomenda-se repetir o esquema completo. 33. Vacinar os profissionais de saúde e os familiares das PVHA é uma forma de ampliar a proteção deste público. 34. O momento ideal para iniciar qualquer vacina para PVHA é seis meses após o início da TARV. 35. O esquema de vacinação contra HPV em PVHA é de três doses (0, 2 e 6 meses) na faixa etária de 9 a 45 anos de idade.

*PVHA - pessoa vivendo com HIV/AIDS; SUS - Sistema Único de Saúde; PNI - Programa Nacional de Imunizações; RNA - ácido ribonucleico; HIV - vírus da imunodeficiência humana; TARV- terapia antirretroviral.*

Os *experts* responderam questões referentes à sua identificação, além de questões referentes à validade do conteúdo da ferramenta. As questões referentes à validade do conteúdo da ferramenta (n=24) foram divididas em conteúdo (n=9), objetivo (n=5), linguagem escrita (n=7) e relevância (n=3). Para cada item, o profissional deveria escolher apenas uma resposta na escala tipo Likert: “concordo totalmente” (5), “concordo” (4), “discordo” (3), “discordo totalmente” (2) ou “não sei”^(16,17)^.

### Análise dos resultados e estatísticas

Na análise para caracterização dos *experts*, foi realizado o cálculo das frequências absolutas (n) e relativas (%) das variáveis categóricas, e da média e do desvio padrão das variáveis numéricas, utilizando o *software Statistical Package for the Social Sciences*, versão 29.

A partir das respostas dos profissionais, foi calculado o Índice de Validade de Conteúdo (IVC) para validação da ferramenta de avaliação do conhecimento. Para tal, considerou-se a porcentagem de *experts* que estavam de acordo com cada item avaliado, tendo como parâmetro de validade um índice de concordância a partir de 80%. Para o cálculo do IVC, foi utilizada a somatória das respostas “concordo totalmente” e “concordo” de cada *expert* em cada item do questionário de validação dividido pelo número total de respostas. Os itens com avaliação “discordo” e “discordo totalmente” foram revistos de acordo com as sugestões dos juízes^(15,18)^.

## RESULTADOS

Para validação da ferramenta de avaliação do conhecimento em imunização de PVHA para profissionais de saúde, foram enviados convites por e-mail para 33 especialistas, com um retorno de 11 especialistas dentro do prazo estipulado de 15 dias (33,3% de adesão).

Em geral, os *experts* eram predominantemente do sexo feminino (81,8%; n=9). A média de idade entre os especialistas que concluíram a etapa de validação (n=11) foi de 53,2 anos (mínimo: 38,6; máximo: 68,9; DP +/- 10,1), variando entre 30 e 69 anos, sendo a maioria nas faixas etárias entre 40 e 49 anos (36,4%; n=4), e entre 60 e 69 anos (36,4%; n=4).

Em relação à formação dos avaliadores, 54,5% (n=6) eram enfermeiros, e 45,5% (n=5) eram médicos. Quanto ao nível máximo de pós-graduação, 9,0% (n=1) tinham especialização, 45,5% (n=5), mestrado, e 45,5% (n=5), doutorado. O tempo médio de formação foi de 29,9 anos (mínimo: 15; máximo: 44; DP +/- 10,1).

Em relação ao IVC, os valores alcançados foram 0,99 para conteúdo, 0,96 para objetivo, 1,00 para linguagem escrita e 0,98 para relevância. Dessa forma, a ferramenta de avaliação do conhecimento foi considerada validada quanto ao seu conteúdo, atingindo um IVC geral de 0,98 (Tabela 1).

**Tabela 1:** Índice de Validade de Conteúdo da ferramenta “Vacina+HIV - avaliação do conhecimento em imunização de pessoas que vivem com HIV/AIDS para profissionais de saúde”, Ribeirão Preto, São Paulo, Brasil, 2025 (N=11)

	Concordo totalmente	Concordo	Discordo	Discordo totalmente	Não sei	IVC
**Conteúdo**						0,99
O conteúdo apresentado na ferramenta de avaliação do conhecimento corresponde aos objetivos propostos.	7	4	0	0	0	1,00
O conteúdo avaliado obedece a uma sequência lógica.	8	3	0	0	0	1,00
Os conteúdos abordados estão de acordo com o recomendado pelo Ministério da Saúde atualmente.	9	2	0	0	0	1,00
Os questionamentos apresentados são apropriados ao público-alvo.	6	5	0	0	0	1,00
O tema da ferramenta de avaliação do conhecimento é atual.	10	1	0	0	0	1,00
O enunciado das questões apresenta-se de forma clara.	8	2	0	1	0	0,91
As questões não apresentam ambiguidades.	8	3	0	0	0	1,00
O número de questões da ferramenta de avaliação do conhecimento está adequado.	7	4	0	0	0	1,00
As questões concentram assuntos relevantes.	10	1	0	0	0	1,00
**Objetivo**						0,96
A aplicação da ferramenta de avaliação do conhecimento é adequada para avaliar o conhecimento de profissionais de saúde em relação à imunização de pessoas que vivem com HIV/AIDS.	4	6	1	0	0	0,91
Os objetivos estão adequados para serem efetivados.	6	5	0	0	0	1,00
A ferramenta de avaliação do conhecimento contempla o tema proposto.	8	3	0	0	0	1,00
A ferramenta de avaliação do conhecimento está adequada ao processo de ensino-aprendizagem.	4	7	0	0	0	1,00
A ferramenta de avaliação do conhecimento proporciona reflexão em relação à vacinação de PVHA.	8	2	1	0	0	0,91
**Linguagem**						1,00
A linguagem utilizada é acessível ao público-alvo.	8	3	0	0	0	1,00
A linguagem é apropriada à ferramenta de avaliação do conhecimento.	10	1	0	0	0	1,00
A linguagem verbal é de fácil assimilação.	6	5	0	0	0	1,00
Os enunciados são abordados de forma clara e objetiva.	8	3	0	0	0	1,00
A ferramenta de avaliação do conhecimento não contém algum erro ou ideia prejudicial em relação às informações.	8	3	0	0	0	1,00
Os enunciados das questões são objetivos.	8	3	0	0	0	1,00
Os enunciados não utilizam termos absolutos (sempre/nunca) ou vagos (raramente/normalmente).	9	2	0	0	0	1,00
**Relevância**						0,98
A ferramenta de avaliação do conhecimento aborda aspectos importantes para o conhecimento dos aspectos envolvidos com a vacinação de PVHA.	9	2	0	0	0	1,00
A ferramenta de avaliação do conhecimento estimula o aprendizado.	7	3	1	0	0	0,91
A ferramenta de avaliação do conhecimento contribui para avaliar o conhecimento em relação à vacinação de PVHA.	8	2	1	0	0	0,94
**IVC geral**						0,98

*IVC - Índice de Validade de Conteúdo; PVHA - pessoas que vivem com HIV/AIDS.*

Em relação ao conteúdo, os especialistas sugeriram que fossem incluídas mais questões discutindo casos práticos, como a questão 21. Optamos por não incluir mais casos práticos por entendermos que as condutas podem ser diferentes de acordo com a localidade e que os casos práticos poderiam ser desenvolvidos pelas equipes durante as atividades de capacitação levando em consideração essas diferenças.

Foi recomendada alteração no texto das questões 9, 10, 20, 21, 32 e 35. Para que o enunciado ficasse mais completo e claro, a maioria das sugestões foi acatada, e os enunciados foram reescritos conforme as sugestões recebidas.

Foi apontado que algumas questões, como as 08, 12, 13 e 14, apresentavam um grau de dificuldade alto para profissionais de nível médio ou que não trabalhassem diretamente com a temática. Como a ferramenta é voltada para aplicação apenas para profissionais que atuam com a temática, e o IVC das questões relacionadas ao conteúdo foi 1,00 quase na totalidade, optamos em manter as questões na forma que foi apresentada aos especialistas.

Ao avaliar o quesito objetivo e relevância da ferramenta, os especialistas apontaram o mesmo como importante e útil, porém ressaltaram que ele deve ser utilizado como parte de atividade educativa, sendo uma das tecnologias utilizadas associadas a outras que propiciem a discussão de casos e de dúvidas.

Os especialistas destacaram a importância de estimular os profissionais a buscar a complementação do conhecimento em outras fontes de informação, além de utilizar outras estratégias em atividades de educação permanente, como estudos de caso, discussões práticas, observação de habilidades ou avaliações formativas, o que permitiria uma análise mais abrangente do conhecimento e das competências dos profissionais envolvidos.

Foi apontada também a importância de se discutir com os profissionais a organização dos CRIE na sua localidade, para que compreendam onde e como os imunobiológicos oferecidos de forma especial podem ser acessados.

As sugestões apresentadas pelos especialistas colaboraram para que a ferramenta de avaliação “Vacina+HIV - avaliação do conhecimento em imunização de pessoas que vivem com HIV/AIDS para profissionais de saúde”, a partir das modificações realizadas, fosse disponibilizada de forma mais clara para uso pelas equipes sem erros na avaliação do conhecimento técnico-científico.

## DISCUSSÃO

As PVHA apresentam um risco maior de complicações e mortes pelas doenças que podem ser prevenidas por vacinas. Por isso, há recomendações especiais de vacinação^(2)^.

Diante da complexidade do calendário de vacinação indicado para este público, a conduta dos profissionais de saúde, que têm um papel essencial na indicação de vacinas e avaliação da completude do esquema vacinal, pode contribuir para o não alcance das taxas de vacinação almejadas.

Estudos apontam que os índices de vacinação para PVHA estão abaixo do esperado para a maioria das vacinas indicadas para este público. As razões para não se vacinar são multifatoriais, desde questões relacionadas ao indivíduo a ser vacinado, aos serviços de saúde e às equipes desses serviços, até a disponibilidade de vacinas^(7,16,19,20)^.

Os profissionais de saúde têm um papel central na avaliação da situação vacinal dos indivíduos e na indicação dos imunizantes de acordo com os protocolos estabelecidos. Como esses protocolos são complexos e sofrem sucessivas modificações, é necessário que os profissionais recebam capacitações constantes^(5)^.

A recomendação da vacinação por profissionais de saúde apresenta uma associação positiva em relação às taxas de vacinação, uma vez que aumenta a conscientização a respeito da importância da indicação dos imunizantes, principalmente para os grupos de maior risco, porém este é um tema pouco abordado na formação dos médicos e equipe de enfermagem^(21)^.

A primeira categoria da ferramenta de avaliação do conhecimento avalia de que forma os profissionais entendem a vacinação como uma importante prática de saúde pública para o controle de doenças.

Nos últimos anos, o sucesso dos programas de imunização, por meio da diminuição e até da erradicação de doenças, contribuiu para que a percepção de risco diminuísse e que aumentasse a insegurança em relação às vacinas, em um cenário onde o medo do evento adverso é maior do que o medo da doença e o que ela pode causar. A hesitação vacinal, apesar de ser um termo recentemente utilizado, vem crescendo em todo o mundo, inclusive entre profissionais de saúde, e deve fazer parte dos conteúdos de capacitação em imunização para estimular as equipes a combaterem-na^(13,22)^.

Os conceitos básicos de imunização são avaliados na segunda categoria da ferramenta de avaliação do conhecimento, na qual são abordados termos complexos, como a definição de vacinas combinadas, conjugadas e polissacarídicas. Temas como esses podem ser desconhecidos ou gerar confusão entre os profissionais de imunização, o que pode levar à falsa contraindicação e à perda de oportunidades de vacinação.

Esses termos precisam ser constantemente reforçados nas atividades de educação permanente, para que sejam absorvidos de forma definitiva entre os profissionais, com um reforço maior, principalmente, entre os profissionais de nível médio, que podem apresentar um grau de dificuldade maior para compreender esses termos e a sua aplicação na prática do trabalho diário durante a triagem em uma sala de vacinação^(5,19,20)^.

A terceira categoria da ferramenta aborda as vacinas atenuadas e inativadas, e suas indicações para PVHA. A administração de vacinas atenuadas é contraindicada na vigência de imunodepressão grave, e este fato pode gerar uma falsa contraindicação de imunizantes às PVHA^(5)^.

Praticamente, a totalidade das PVHA em TARV com boa adesão atinge a supressão viral, não estando, assim, em imunodepressão. Portanto, não apresentam contraindicação para receber os imunizantes com agentes vivos em sua composição. Por conta disso, as equipes precisam estar seguras para administrar esses imunizantes, e a prescrição médica auxilia de forma contundente a garantir esta segurança^(5,20)^.

Trabalhar com o tema imunização de PVHA entre profissionais de saúde exige que estes tenham bem estabelecido em seu arcabouço de conhecimento, além de conceitos relacionados à imunologia, a diferença entre viver com HIV e ser imunossuprimido.

É necessário retomar os conceitos básicos do que é o HIV, imunodepressão e TARV, para que, posteriormente, consigam dar continuidade à integralidade do cuidado que inclui o esquema de vacinação.

A última categoria da ferramenta de avaliação do conhecimento aborda o esquema vacinal para adultos infectados pelo HIV, discutindo esquemas de algumas vacinas indicadas e o melhor período para administrar as vacinas, além da prevenção através da vacinação de contatos domiciliares.

As questões não esgotam o tema. É necessário que, durante as atividades de educação permanente, todas as vacinas e seus esquemas, as situações de contraindicações e especificidades de cada localidade (onde as vacinas são oferecidas e de que forma podem ser acessadas) sejam amplamente abordadas.

Ao utilizar a ferramenta validada neste estudo, sugerimos que o conhecimento na temática seja avaliado pelo percentual de acertos por intervalo de 20% (insuficiente (0 -| 20); regular (20 -| 40); bom (40 -| 60); ótimo (60 -| 80); excelente (80 -| 100)), conforme proposto por outros autores^(23)^.

### Limitações do estudo

Entendemos que a ferramenta de avaliação do conhecimento utilizada de forma isolada, fora de atividades educativas, pode não ser eficaz para aumentar o conhecimento dos profissionais em relação à imunização de PVHA; por isso, indicamos que ela seja utilizada como parte dessas atividades, para avaliar o conhecimento prévio dos profissionais.

Além disso, poderá ser utilizada após uma atividade de capacitação para avaliar se o objetivo foi alcançado.

Outra limitação do estudo é o fato de a amostra de *experts* ser relativamente pequena (n=11) e por conveniência, o que pode gerar um viés nos resultados do estudo. Apesar disso, entendemos que a ferramenta validada neste estudo não deixa de ser um instrumento que pode auxiliar as equipes nas atividades de educação permanente nesta temática, identificando lacunas no conhecimento que precisam ser melhoradas.

### Contribuições para as áreas da enfermagem, saúde ou políticas públicas

Os resultados apresentados neste estudo contribuem para disponibilizar para as equipes uma ferramenta que poderá ser utilizada nas atividades de educação permanente em saúde, contribuindo para identificar lacunas no conhecimento que podem ser melhor abordadas em diferentes estratégias de capacitação. Todos os itens avaliados foram considerados “totalmente adequado” ou “adequado” pela maioria dos especialistas, demonstrando que o objetivo de atualizar e validar uma ferramenta de avaliação do conhecimento para disponibilizá-lo para uso em atividades educativas em todo o país foi atingido. A ferramenta desenvolvida e validada neste estudo é uma das tecnologias que poderá ser utilizada durante a capacitação dos profissionais de saúde nesta temática, contribuindo também para melhoria das taxas de vacinação em PVHA e, consequentemente, melhoria da qualidade e expectativa de vida dessas pessoas.

Esta ferramenta validada poderá ser utilizada pelas equipes dos serviços de saúde com sala de vacinação e nos serviços de saúde que realizam o atendimento de PVHA para identificar as lacunas no conhecimento dos profissionais desses serviços.

A partir da identificação dessas lacunas, ações de educação permanente das equipes podem ser desenvolvidas para melhorar o conhecimento dos profissionais a respeito dessa temática, o que poderá auxiliar diretamente para aumentar as taxas de vacinação de PVHA.

## CONCLUSÕES

Destaca-se a relevância deste estudo, uma vez que nenhuma ferramenta de avaliação do conhecimento validada sobre imunização de PVHA foi encontrada na literatura. Assim, acredita-se que esta seja a primeira ferramenta de avaliação do conhecimento validada sobre o tema voltado para profissionais de saúde no Brasil.

Concluímos que a ferramenta “Vacina+HIV - avaliação do conhecimento em imunização de pessoas que vivem com HIV/AIDS para profissionais de saúde” poderá auxiliar as equipes envolvidas nas atividades de educação permanente e que conteúdos não abordados e questões relacionadas à prática de cada localidade devem ser incluídos em outras estratégias educativas utilizadas.

## Data Availability

Os dados de pesquisa estão disponíveis no corpo do artigo.
